# Determination of blood sirolimus concentrations in liver and kidney transplant recipients using the Innofluor® fluorescence polarization immunoassay: Comparison with the microparticle enzyme immunoassay and high-performance liquid chromatography-ultraviolet method

**DOI:** 10.1080/03009730802608254

**Published:** 2009-02-04

**Authors:** Lorena Bouzas, Jesús Hermida, J Carlos Tutor

**Affiliations:** Unidad Monitorización Fármacos, Laboratorio Central, Hospital Clínico Universitario, Santiago de CompostelaSpain

**Keywords:** Fluorescence polarization immunoassay, high-performance liquid chromatography, microparticle enzyme immunoassay, sirolimus

## Abstract

**Background:**

Although high-performance liquid chromatography (HPLC) is the method of choice for blood sirolimus determination, the microparticle enzyme immunoassay (MEIA) run on the IMx® analyser is widely used in therapeutic monitoring of this immunosuppressant agent. The aim of our study was to evaluate the possible determination of sirolimus using the fluorescence polarization immunoassay (FPIA) commercialized for everolimus quantification.

**Methods:**

Sirolimus concentrations were determined in whole-blood samples from liver and kidney transplant recipients using the Innofluor® Certican® FPIA (Seradyn Inc.) run on a TDx® analyser (Abbott Laboratories), Sirolimus MEIA run on an IMx® analyser (Abbott Laboratories), and HPLC (UV detection) methods.

**Results:**

The Innofluor® FPIA has a similar cross-reactivity with everolimus and sirolimus, and the within- and between-run coefficients of variation obtained for sirolimus determination were 2.7%–13.3%. In analysing different blood samples from liver and kidney transplant patients the linear regressions obtained were: FPIA = 1.12 HPLC + 0.43 (*n*=104, *r*=0.874), MEIA = 1.14 HPLC (*n*=146, *r*=0.892), and FPIA = 1.00 MEIA + 0.29 (*n*=106, *r*=0.941). Better correlation coefficients were obtained between the methods in the liver transplant samples (*r*≥0.900) than in the kidney transplant samples (*r*≥0.849). No significant effect was found for sirolimus clearance or the blood hematocrit on the relationship between the results produced by both immunoassays and HPLC.

**Conclusion:**

The Innofluor® FPIA is a valid alternative with an analogous performance to the MEIA for the therapeutic monitoring of sirolimus.

## Introduction

Sirolimus (Rapamune®, Wyeth-Ayerst, Princeton, USA) is a macrolide antibiotic with potent antiproliferation and immunosuppressant properties. The drug has a narrow therapeutic index, and a poor correlation between the doses and blood concentrations, resulting from the variability in metabolism and P glycoprotein drug transport mechanisms (1). Clinical studies have shown significant correlations between trough sirolimus concentrations and the area under the concentration-time curve, with a strong relationship between drug toxicity and trough concentrations above 15 µg/L (2,3). Consequently, therapeutic monitoring of sirolimus is necessary in order to minimize adverse side-effects and to ensure effective immunosuppression. As 94% of sirolimus is found within erythrocytes, EDTA-anticoagulated whole blood is the appropriate matrix for its quantification (3).

The recommended and still most commonly used method for blood sirolimus determination is high-performance liquid chromatography (HPLC), using tandem mass spectrometric detection or ultraviolet detection. However, simpler techniques for sirolimus management have been developed, such as the microparticle enzyme immunoassay (MEIA) or the cloned enzyme donor immunoassay (CEDIA). Sirolimus measurements using HPLC have tended to be focused in larger transplant centres, although sirolimus immunoassays may be performed by local laboratories providing a more rapid turn-around time of results (4). The MEIA assay has been extensively evaluated, and in accordance with the majority of authors is a viable alternative to HPLC-based methods for routine sirolimus monitoring (4–12); however, due to the sirolimus-metabolite cross-reactivity with the antibody, users must consider the implications of the variable MEIA overestimation when interpreting the results (13). With respect to the sirolimus CEDIA, few studies on its analytical performance have been published, and they reached discordant conclusions (14,15).

A fluorescence polarization immunoassay (FPIA) is marketed by Seradyn Inc. for the determination of everolimus (Certican®, Novartis Pharma AG, Basel, Switzerland), an immunosuppressive derivative of sirolimus with a 2-hydroxyethyl chain at position 40 (16). As sirolimus and everolimus molecules are highly similar, and an analogous cross-reactivity against the antibody may be expected, the present study was aimed at evaluating whether the everolimus FPIA assay could be applied to the determination of sirolimus. The study includes correlations with the MEIA and HPLC/UV.

## Material and methods

The sirolimus levels were determined in whole-blood samples from different adult liver and kidney transplant recipients, both in the immediate post-transplant period and the maintenance period. In most cases, sirolimus was administered in combination with low-dose calcineurin inhibitors: tacrolimus in liver recipients and tacrolimus or cyclosporin in kidney recipients. The samples were taken in Vacutainer® tubes containing EDTA prior to the next dose of sirolimus, and at least after a 10-day period without any modification of the dosage. As a result, the blood sirolimus concentrations correspond to the steady-state trough levels. The study was carried out according to the good practice rules for investigation in humans of the Conselleria de Sanidade (Regional Ministry of Health) of the Xunta de Galicia, Santiago de Compostela, Spain.

Sirolimus determination using the Innofluor® Certican® FPIA (Seradyn Inc., Indianapolis, USA) was carried out according to the manufacturer's specifications for everolimus determination, using a TDx® analyser (Abbott Laboratories, Abbott Park, USA) and calibrated with Innofluor® Certican® calibrators (Seradyn Inc.). The determinations using the Sirolimus MEIA (Abbott Laboratories) were carried out according to the manufacturer's instructions in an IMx® analyser (Abbott Laboratories). The blood concentrations of sirolimus were also determined by HPLC with UV detection in an Agilent 1200 series system, using a slightly modified version of the method of French et al. (17), in which after 1-chlorobutane extraction the supernatant was dried using a SPD1010 SpeedVac® system (Thermo Savant). The IMx® Sirolimus controls (Abbott Laboratories) were used for the intralaboratory daily quality control. Sirolimus clearance (CL) was estimated using the equation (18): CL=(F)(Dose/ℸ)/Css, where F corresponds to the sirolimus bioavailability (approximately 0.15), ℸ is the dosing interval, and Css the whole-blood sirolimus concentration determined by HPLC. As trough rather than average sirolimus concentrations were used, reported CL represents overestimates of the actual values. The serum levels of albumin, bilirubin, creatinine, and urea were determined in an Advia 2400 Chemistry System. The glomerular filtration rate (GFR) was estimated from age, sex, race, and serum creatinine, albumin, and urea concentrations, using the 6-variable Modification of Diet in Renal Disease formula (19).

Statistical analysis was carried out using the StatGraphics Plus (v. 5.0) package, and the Shapiro-Wilks method was used to check the distribution of data. Pearson's correlation coefficient was used when the data had a Gaussian distribution; otherwise, Spearman's correlation coefficient was used. The regression analysis was made using the Passing-Bablok non-parametric method, and consequently the ma68 value was used as standard error of the estimate for the evaluation of dispersion data. The results obtained with the different methods were also compared using Eksborg difference plots (20). In accordance with previously established criteria (21,22), and considering a therapeutic range for sirolimus of 5–15 µg/L (1,3), the clinically acceptable deviation error is ≤ 1.3 µg/L, with an acceptable value ≤ 0.6 µ/L for the standard error of the estimate, and a clinically acceptable coefficient of variation ≤ 6.3%. Similarly, in accordance with the consensus criteria for the validation of analytical methods used for the quantitative determination of drugs and their metabolites in biological samples (23,24), the accepted level for accuracy is a deviation error of no more than 15% from the nominal value, and for imprecision a variation coefficient no higher than 15% (for levels above the limit of quantification). The results were expressed as mean±SD (median).

## Results

Our MEIA and HPLC assays fulfilled the acceptance criteria of the Sirolimus International Proficiency Testing Scheme survey, which includes three monthly pooled blood samples from patients receiving sirolimus, or blood samples with added sirolimus. For 59 samples, the mean of the monthly overall laboratory method means were 13.8±10.99 µg/L (median 9.6 µg/L) for HPLC/UV and 12.9±10.31 µg/L (median 9.3 µg/L) for MEIA. In our laboratory, the mean sirolimus concentration obtained for the same 59 samples using the HPLC/UV assay was 12.4±9.67 µg/L (median 9.1 µg/L), and using MEIA 12.2±9.37 µg/L (median 9.0 µg/L). The correlation coefficients obtained between our individual 59 results and the overall laboratory method means were *r*=0.950 (*P* <0.001) for HPLC, and *r*=0.965 (*P* <0.001) for MEIA. The mean sirolimus concentration obtained for these 59 interlaboratory quality control samples using the FPIA assay was 12.8±9.36 µg/L (median 9.7 µg/L). The Innofluor® FPIA assay had practically a sirolimus cross-reactivity of 100%, significantly greater than the value of 72% indicated in the package insert, with lower limits of detection and quantitation respectively about 1.0 µg/L and 2.0 µg/L.

The within-run imprecision study was carried out using the duplicates method, with blood samples from liver and kidney transplant recipients assayed twice for each of the methods, and with sirolimus concentrations <5 µg/L, 5–10 µg/L and >10 µg/L. Using the Innofluor® FPIA for 10 duplicates with a mean sirolimus concentration of 3.8±0.25 µg/L, a variation coefficient of 6.6% was obtained, for 41 duplicates with a mean concentration of 7.4±0.46 µg/L a variation coefficient of 6.3%, and for 13 duplicates with a mean concentration of 14.2±0.39 µg/L a variation coefficient of 2.7%. Using the MEIA for 32 duplicates with a mean concentration of 3.5±0.20 µg/L a variation coefficient of 5.5% was obtained, for 111 duplicates with a mean concentration of 7.6±0.28 µg/L a variation coefficient of 3.7%, and for 51 duplicates with a mean concentration of 16.0±0.54 µg/L a variation coefficient of 3.4%. Using the HPLC method for 40 duplicates with a mean concentration of 3.8±0.34 µg/L a variation coefficient of 9.0% was obtained, for 98 duplicates with a mean concentration of 7.2±0.39 µg/L a variation coefficient of 5.5%, and for 41 duplicates with a mean concentration of 17.2±0.67 µg/L a variation coefficient of 3.9%. Between-run imprecision for the different methods was studied in the daily quality control using the IMx® Sirolimus controls, and the results are shown in Table I. For the FPIA assay an analogous between-run imprecision was obtained using the Innofluor® Certican® controls (data not shown). In some cases, the obtained variation coefficients were higher than the clinically acceptable value of 6.3% according to previously published criteria (21); however, in all cases these variation coefficients (<15%) were acceptable in accordance with the consensus recommendations for the validation of analytical methods for determination of drugs and their metabolites (1,23).

**Table I.** Between-run imprecision study for sirolimus determination.

	*n*	Mean±SD (µg/L)	CV (%)
Innofluor® FPIA			
IMx® Sirolimus control L	30	5.41±0.72	13.3
IMx® Sirolimus control M	30	10.58±1.02	9.7
IMx® Sirolimus control H	30	21.03±2.08	9.9
MEIA			
IMx® Sirolimus control L	100	4.80±0.41	8.6
IMx® Sirolimus control M	100	10.30±0.69	6.7
IMx® Sirolimus control H	100	22.28±1.56	7.0
HPLC			
IMx® Sirolimus control L	22	4.96±0.59	11.9
IMx® Sirolimus control M	22	11.09±0.81	7.3
IMx® Sirolimus control H	22	22.50±1.98	8.8

Assigned values: control L = 5.0 µg/L; control M = 11.0 µg/L; control H = 22.0 µg/L.

**Figure 1.**
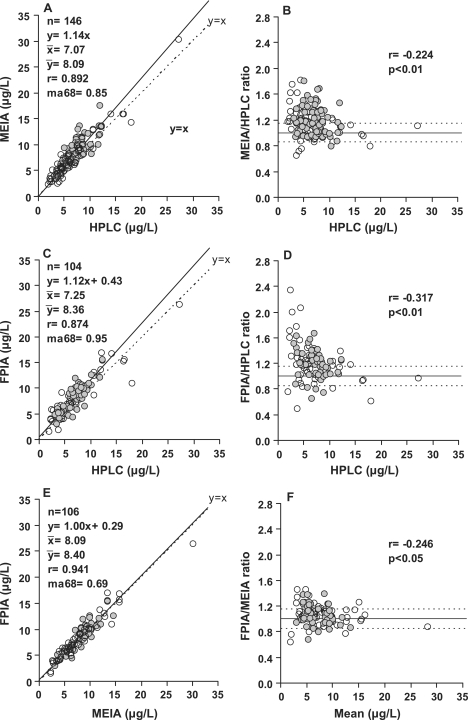
Correlation and regression (A, C, E) and difference plots (B, D, F) for sirolimus concentrations using the MEIA, FPIA, and HPLC in blood samples from liver (○) and kidney (•) transplant recipient patients. The dotted lines correspond to the limits of the acceptance criterion for deviation.

The regression obtained between the MEIA and HPLC results for the total number of samples considered (Figure 1A) was in line with previously published results (4–6,9,11,13). The differences between the means (medians) obtained using the FPIA, MEIA, and HPLC methods were lower than the acceptable deviation error in accordance with the considered validation criteria (1,21–23); however, the standard errors of the estimates were slightly higher than the clinically acceptable value (0.6 µg/L) according to the considered criterion (21) as is shown in Figure 1 (A, C, E). Similarly, the difference plots (Figure 1B, D) show that a considerable number of individual results provided by MEIA and FPIA differ with respect to the HPLC (nominal) values by more than 15%.

In the liver transplant recipients, the regression and correlation between FPIA and HPLC results were: FPIA = 1.09 HPLC + 0.57 (*n*=54, *r*=0.900, ma68 = 1.0 µg/L), and in renal transplant patients: FPIA = 1.18 HPLC + 0.04 (*n* =50, *r* =0.772, ma68 = 0.95 µg/L). The relationship between MEIA and HPLC in the liver transplant recipients was: MEIA = 1.08 HPLC + 0.31 (*n*=62, *r*=0.925, ma68 = 0.77 µg/L), and in the renal recipients: MEIA = 1.24 HPLC – 0.63 (*n*=84, *r*=0.764, ma68 = 0.90 µg/L). The regression and correlation obtained between the FPIA and MEIA in the liver transplant recipients were: FPIA = 1.00 MEIA + 0.37 (*n*=54, *r*=0.964, ma68 = 0.72 µg/L), and in the renal transplant recipients: FPIA = 1.04 MEIA – 0.21 (*n*=52, *r*=0.849, ma68 = 0.70 µg/L).

The group of liver transplant patients had significantly higher levels of GFR (*P* <0.001), and bilirubin (*P* <0.05), and significantly lower levels of albumin (*P* <0.001), than the group of renal transplant patients. As shown in Table II, no significant differences were found between both groups of patients for sirolimus CL, and the FPIA/HPLC, MEIA/HPLC, and FPIA/MEIA sirolimus concentration ratios. The interindividual variation for sirolimus CL was greater in the group of liver transplant recipients (CV = 74%), than in the kidney transplant recipients group (CV = 40%). Possible pharmacokinetic interactions due to the concomitant administration in some cases of enzyme-inducing or -inhibiting drugs were not considered.

**Table II.** Sirolimus clearance (CL) and sirolimus concentration ratios in liver and kidney transplant recipients.

	Liver transplant (*n*=54)	Kidney transplant (*n*=50)
Sirolimus CL (L/h) ^a^	3.70±2.73 (2.61)	3.46±1.37 (3.19)
FPIA/HPLC ratio	1.21±0.36 (1.18)	1.19±0.21 (1.19)
MEIA/HPLC ratio	1.14±0.23 (1.14)	1.18±0.18 (1.15)
FPIA/MEIA ratio	1.03±0.14 (1.06)	1.03±0.14 (1.02)

^a^Calculated from Css determined by HPLC.

A significant negative correlation was found for sirolimus CL with the sirolimus concentration in the total patient group (*r*= − 0.589, *P* <0.001), kidney transplant patients (*r*= − 0.292, *P* <0.05), and liver transplant patients (*r*= − 0.859, *P* <0.001). Significant positive correlations were also found for the FPIA/HPLC and MEIA/HPLC sirolimus concentration ratios with sirolimus CL in the total patient group (*P* <0.01) and liver transplant patient group (*P* <0.005); however, in the first-order partial correlation, keeping the sirolimus concentration constant, no statistical significance was achieved between these variables. Neither were any significant correlations found in the total patient group for the FPIA/HPLC, MEIA/HPLC, and FPIA/MEIA sirolimus concentration ratios with the estimated GFR (range 19.8–125.3 mL/min/1.73 m^2^), blood hematocrit (range 20.7%–48.5%), albumin (range 1.5–4.4 g/dL), or bilirubin (range 0.1–2.2 mg/dL).

## Discussion

The results obtained in the imprecision study for FPIA, MEIA, and HPLC may be considered satisfactory. Although the variation coefficients in some cases were higher than the clinically acceptable value of 6.3% (21), the acceptance limit of 15% (23,24) was not exceeded in any case. The FPIA imprecision was in general slightly greater than that given by the MEIA assay, and this fact may be due to the FPIA procedure for preparing blood samples, which requires methanol extraction prior to the addition of the precipitation reagent, whereas MEIA uses a simpler procedure with only one extraction/precipitation reagent.

Although some authors have found similar results using MEIA and HPLC (7,8,10,12), it is generally accepted that the MEIA leads to an overestimation of the sirolimus concentration in patients’ samples, related to the antibody reacting with hydroxyl- and desmethyl-sirolimus metabolites (4–6,9,11,13,25). The FPIA and MEIA assays provided similar results, with a high correlation coefficient between them, and a standard error of the estimate only slightly higher than the acceptable value (Figure 1E). Likewise, as shown in Figure 1, both immunoassays tend to overestimate the concentrations of sirolimus with respect to the values obtained by HPLC; however, the difference between the means (medians) obtained is acceptable in accordance with the validation criteria considered (1,21–23). Higher correlation coefficients between the different methods were obtained in the group of liver transplant patients than in the group of kidney transplant patients, although the lower range of sirolimus concentrations in this patient group may be considered a contributing factor to this result.

It has been indicated that other variables, such as the time after kidney transplantation (25) and blood hematocrit (4,25), may be significant in the overestimation of sirolimus by MEIA; however, for other authors these factors do not have any influence on the overestimation of sirolimus using the MEIA assay (6). In our study, it was not possible to confirm the possible interference effect of the hematocrit (range 20.7%–48.5%) on either of the immunoassays used, as statistical significance was not achieved in the correlation of the FPIA/HPLC, MEIA/HPLC, and FPIA/MEIA sirolimus concentration ratios with the hematocrit. Similarly, no significant correlations were found between the sirolimus concentration ratios and the estimated GFR, or the serum levels of albumin and bilirubin.

Variations in sirolimus absorption and clearance result in a wide range of trough concentrations among patients receiving the same dose. Although in hepatic dysfunction the intestinal absorption of sirolimus may not be significantly affected, patients with liver disease showed decreased sirolimus CL (26,27), and increased concentrations of the parent drug and its metabolites (3); however, a considerable overlap in sirolimus CL among healthy subjects and patients with mild, moderate, or severe hepatic impairment was previously described (26,27). In our study, a large overlap was also found between the values of sirolimus CL for the groups of patients with liver and kidney transplants, without any significant difference for this pharmacokinetic variable between both groups. Neither were any significant differences found between these groups of patients for the FPIA/HPLC, MEIA/HPLC, and FPIA/MEIA sirolimus concentration ratios, nor any significant first-order partial correlations for these concentration ratios with sirolimus CL. These results are the opposite to those that would be expected, and suggest that the sirolimus metabolization rate does not appear to be capable of introducing an additional source for the FPIA and MEIA deviation with respect to the HPLC results, whose clinical significance is mainly conditioned by the relative proportions of sirolimus and its metabolites in the blood samples.

The sirolimus-metabolite cross-reactivity with the MEIA antibody could lead to a variable bias with respect to HPLC results; however, this immunoassay is widely used for routine sirolimus determination, with MEIA users currently representing 39% of the total participants in the Sirolimus International Proficiency Testing Scheme survey. In accordance with our results (Figure 1F), a higher cross-reactivity of the Innofluor® FPIA for lower sirolimus concentrations than 5 µg/L has been previously described (28); however, a correction of the results appears unnecessary in the routine practice, and the FPIA assay may be a valid alternative to the MEIA with analogous performance for the therapeutic monitoring of sirolimus.

We have previously described that everolimus may be quantified using the Sirolimus MEIA assay (29), and consequently sirolimus and everolimus determination may be carried out using a common FPIA or MEIA assay, which would reduce the reagent costs. The choice of the Innofluor® Certican® FPIA or the Sirolimus MEIA for the determination of both immunosuppressive agents may be made on the basis of the TDx and IMx analyser availability.
